# Increase in suicides the months after the death of Robin Williams in the US

**DOI:** 10.1371/journal.pone.0191405

**Published:** 2018-02-07

**Authors:** David S. Fink, Julian Santaella-Tenorio, Katherine M. Keyes

**Affiliations:** Department of Epidemiology, Columbia University, New York, New York, United States of America; University of Queensland, AUSTRALIA

## Abstract

Investigating suicides following the death of Robin Williams, a beloved actor and comedian, on August 11^th^, 2014, we used time-series analysis to estimate the expected number of suicides during the months following Williams’ death. Monthly suicide count data in the US (1999–2015) were from the Centers for Disease Control and Prevention Wide-ranging ONline Data for Epidemiologic Research (CDC WONDER). Expected suicides were calculated using a seasonal autoregressive integrated moving averages model to account for both the seasonal patterns and autoregression. Time-series models indicated that we would expect 16,849 suicides from August to December 2014; however, we observed 18,690 suicides in that period, suggesting an excess of 1,841 cases (9.85% increase). Although excess suicides were observed across gender and age groups, males and persons aged 30–44 had the greatest increase in excess suicide events. This study documents associations between Robin Williams’ death and suicide deaths in the population thereafter.

## Introduction

On August 11, 2014, well-known actor and comedian Robin Williams committed suicide in his home in Paradise Cay, California at the age of 63. The details of his suicide were widely reported in the media in the days and weeks that followed. Although Williams’ widow revealed his struggles with Lewy Body Dementia, the initial reports on Williams’ death did not mention this condition.

The effects of a widely reported celebrity suicide on population health are extensively documented in the international literature [1–9]. Recent meta-analysis suggests that, on average, suicides increase by approximately 0.26 per 100,000 population in the weeks following a high profile celebrity suicide [1], with heterogeneity in the magnitude of the effect across country, event, and celebrity. Effects are particularly prominent when the celebrity is an entertainment star [2, 3], among other factors. While some evidence indicates that suicides in the population tend to increase among those at a similar age as the deceased and using a similar method [4, 10, 11], such evidence is not always consistent [12]. However, there is a paucity of evidence about the effects of a celebrity suicide on population health within the U.S. [13], and no study, to our knowledge, has examined this effect within the modern era of the 24-hours news cycle.

Celebrity suicide effects have led to the World Health Organization to establishment media guidelines for reporting a high profile celebrity death, including sensitivity and non-sensationalism in the reporting of the means of suicide, the precipitating factors, and the risk factors for suicide apparent in the deceased [14], and clear and consistent messages about suicide prevention and help-seeking during reporting [6, 15]. The extent to which these guidelines were followed after the death of Mr. Williams, however, is questionable, and as such, we examined suicide incidence in the United States by month surrounding the time frame of Mr. Williams’ death.

## Materials and methods

### Data sources

Monthly suicide count data and monthly suicide rates (from January 1999 to December 2015), by sex, age, and method, in the United States were from the Centers for Disease Control and Prevention Wide-ranging ONline Data for Epidemiologic Research (CDC WONDER) [16]. These are publicly available, de-identified, data exempt from review by an institutional review board.

We also used the Bloomberg Terminal’s (http://www.bloomberg.com/professional/education/) news trend (NT) function to identify the number of global English-language news media reports with first, the terms *Suicide* and *Dead*, and second, the term *Robin Williams*. To provide a comparison to the same time during the previous year, we analyzed the number of news media reports from June 1, 2013 to January 1, 2015.

### Analyses

Time-series analysis was used to determine the expected number of suicides during the months following Williams’ death. Over the calendar year, the monthly number of suicides in a population tends to exhibit a seasonal pattern, with a peak in the early spring and trough in the summer months. As a result, the error term of these observations are often autocorrelated, and not independent, which violates an assumption of ordinary least squares regression. Time-series analysis accounts for the violations of the independence assumption and models the data generating process. Therefore, we used a seasonal autoregressive integrated moving averages (SARIMA) model for this study [17]. Seasonal variation and linear trends were removed by differencing the population parameters between successive years and months, respectively. We examined both the autocorrelation function and partial autocorrelation function to identify the best fitting autoregressive and moving average parameters. The model that provided the lowest Akaike information criterion and Bayesian information Criterion was chosen to best model the data generation process. To assess the difference in observed suicides and expected suicides following Williams’ death on August 14^th^, 2014, we used an SARIMA(0,1,1)x(0,1,1)12 to model the observed number of suicides from January 1999 to July 2014 and forecasted the expected number of suicides, and 95% confidence intervals (CIs), from August 2014 to December 2014. There was no evidence that residuals deviated from white noise (Q tests, p = 0.31). All analyses were performed using R version 3.2.4 (Vienna, Austria) and STATA version 14 (College Station, Texas).

## Results

**Fig 1:**
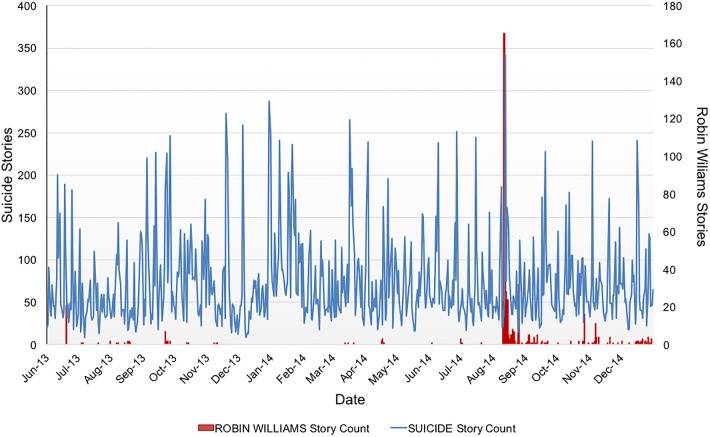
News reports related to “Suicide” and “Robin Williams” from June 2013 through December 2014. Number of suicide stories are presented for each month from June 2013 to December 2014 (light blue line). Number of Robin Williams stories for each month from June 2013 to December 2014 (red bars).

News media reports with the terms *Suicide* and *Dead*, and the term *Robin Williams* drastically increased in the weeks after Mr. Williams’ death (Fig 1). These increments in media reports were greater that those observed in previous months and also during the August-December period in 2013. Although the maximum number of stories about Robin Williams was during the approximately four weeks after his death from August-September 2014, stories about Williams were published during the entire period from August-December 2014.

**Fig 2:**
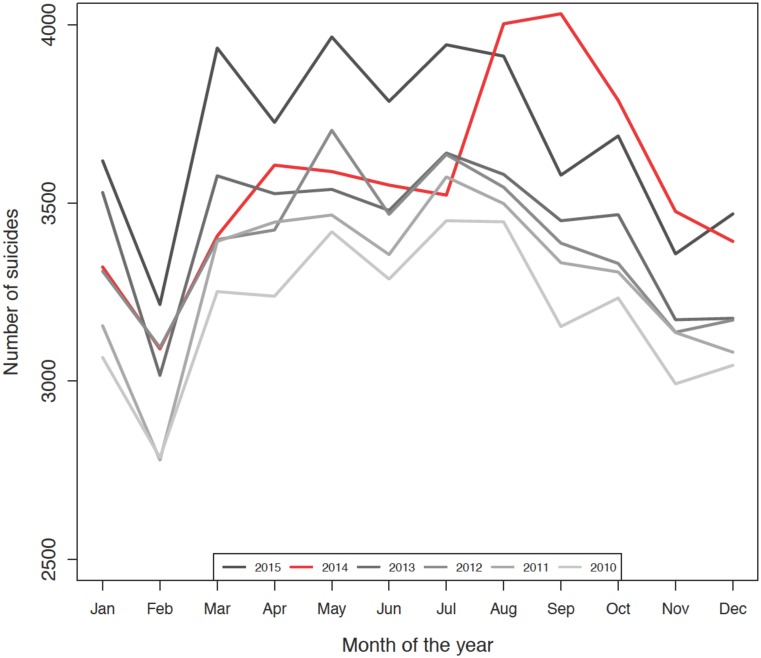
Monthly number of suicides in the United States from January 2010 to December 2015. Number of suicides are presented for each month, January to December, from 2010 (lightest grey) to 2015 (darkest grey). The year of Robin Williams’ suicide is in red (2014).

Fig 2 shows the number of suicides in the United States by month from 2010 through 2015. Monthly suicide counts followed a consistent seasonal pattern over the year, with the fewest suicides occurring in February and counts increasing over the summer months. Furthermore, monthly suicide counts tended to increase each year over the prior year’s count for that same month such that more suicides were recorded for January 2011 than January 2010. Compared to prior years, there was a marked increase in the number of suicides beginning in August 2014 through December 2014. This increase in the number of suicides per month appeared to remain consistent over 2015.

**Fig 3:**
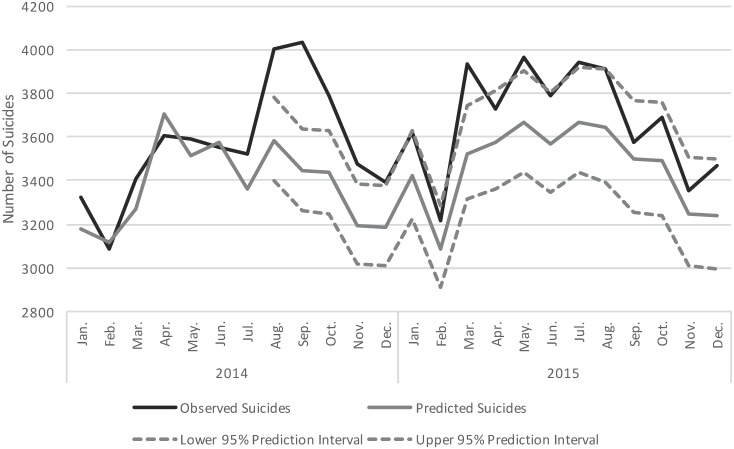
Number of observed and predicted suicide deaths in the United States from January 2014 to December 2015. Number of observed suicide deaths (black line) and number of predicted suicide deaths (grey line) with 95% prediction intervals (dashed grey lines) are presented. The 95% prediction intervals are presented for the months following the death of Robin Williams in August, 2014.

Fig 3 shows the observed and predicted number of suicides in the months that followed Williams’ death. Specifically, we observed a 9.9% increase in the number of suicides in the US from August to December 2014. The monthly number of suicides observed during 2015 followed the upper 95% prediction interval.

Increases in suicides from August to December 2014 were consistently observed across gender, both in males (9.1%) and females (9.7%), and across all age groups; those ages 30–44 showed the greatest increase in suicides (12.9%) (Table 1). These increments represent and excess of 1,841 cases (1,398 among males, 386 among females, and 175, 265, 577, 363 and 376 cases among those 12–19, 20–29, 30–44, 45–59 and 60+, respectively). Finally, Table 2 show that, compared to all other methods of suicide, the difference between the observed and predicted number of suicides was greatest and most consistent among suffocation suicides. Specifically, we observed a 32.3% increase in the number of suffocation suicides in the five months that followed Williams’ death, compared to a 3.1% increase in the number of suicides from all other methods combined (i.e., cutting/piercing, poisoning, firearm, falls, all other methods), albeit we found a higher than expected number of poisoning suicides and firearm suicides in August and September, respectively.

**Table 1:** Observed and expected number of suicides from August 2014 to December 2014 in the United States.

Demographics	No. of Observed Suicides	Predicted Suicides		Difference between Observed and Predicted Suicides	
		No. of Predicted Suicides	95% PI	Difference	95% PI
Ages 12–19 years					
August	210	159	134, 189	51	21, 76
September	218	178	149, 212	40	6, 69
October	213	186	155, 222	2	-9, 58
November	208	168	140, 202	40	6, 68
December	167	151	125, 182	16	-15, 42
Aug-Dec	1,016	841		175	
Ages 20–29 years					
August	576	549	494, 610	27	-34, 82
September	596	525	472, 585	71	11, 124
October	586	534	479, 596	52	-10, 107
November	570	495	443, 552	75	18, 127
December	536	496	444, 555	40	-19, 92
Aug-Dec	2,864	2,600		264	
Ages 30–44 years					
August	995	841	770, 919	154	76, 225
September	941	784	717, 857	157	84, 224
October	927	789	721, 863	138	64, 206
November	790	749	684, 820	41	-30, 106
December	822	735	670, 805	87	17, 151
Aug-Dec	4,475	3,898		577	
Ages 45–59 years					
August	1,243	1,121	1029, 1221	122	22, 214
September	1,231	1,080	989, 1180	151	51, 242
October	1,073	1,054	963, 1154	19	-81, 110
November	1,020	978	891, 1073	42	-53, 129
December	1,015	986	897, 1084	29	-69, 119
Aug-Dec	5,582	5,219		363	
Ages 60+ years					
August	971	916	827, 1014	55	-43, 144
September	1,039	888	800, 984	151	55, 238
October	985	883	796, 980	102	5, 189
November	881	832	749, 924	49	-43, 132
December	847	829	745, 921	18	-74, 102
Aug-Dec	4,723	4,347		376	
Females					
August	929	804	732, 883	125	46, 197
September	907	782	711, 860	125	47, 196
October	867	802	727, 883	65	-16, 140
November	818	743	673, 820	75	-2, 145
December	706	710	642, 785	-4	-79, 64
Aug-Dec	4,227	3,841		386	
Males					
August	3,074	2,796	2633, 2969	278	105, 441
September	3,124	2,663	2506, 2831	461	293, 618
October	2,921	2,651	2492, 2821	270	100, 429
November	2,658	2,470	2319, 2630	188	28, 339
December	2,686	2,485	2332, 2648	201	38, 354
Aug-Dec	14,463	13,065		1,398	
Total Population					
August	4,003	3,586	3399, 3782	417	221, 604
September	4,031	3,445	3262, 3639	586	392, 769
October	3,788	3,434	3248, 3632	354	156, 54
November	3,476	3,195	3018, 3383	281	93, 458
December	3,392	3,188	3008, 3379	2034	13, 384
Aug-Dec	18,690	16,849		1,841	

Abbreviations: PI, prediction interval

**Table 2:** Observed and expected number of suicides by method from August 2014 to December 2014 in the United States.

Suicide Method	No. of Observed Suicides	Predicted Suicides		Difference between Observed and Predicted Suicides	
		No. of Predicted Suicides	95% PI	Difference	95% PI
Suffocation					
August	1,237	909	814, 1,014	51	21, 76
September	1,266	881	788, 985	40	6, 69
October	1,144	862	770, 965	27	-9, 58
November	929	764	681, 856	40	6, 68
December	971	778	693, 874	16	-15, 42
Aug-Dec	5,547	4,194		175	
Cut/pierce					
August	60	66	48, 90	-6	-30, 12
September	69	65	47, 89	4	-20, 22
October	59	62	45, 86	-3	-27, 14
November	67	60	43, 83	7	-16, 24
December	60	60	43, 83	-0	-23, 17
Aug-Dec	315	312		3	
Poisoning					
August	646	573	510, 643	73	3, 136
September	582	549	488, 617	33	-35, 94
October	557	549	488, 618	8	-61, 69
November	563	538	477, 606	25	-43, 86
December	485	517	459, 584	-32	-99, 26
Aug-Dec	2,833	2,726		107	
Firearm					
August	1,839	1,822	1,703, 1,948	17	-109, 136
September	1,898	1,742	1,627, 1,865	156	33, 271
October	1,819	1,776	1,658, 1,904	43	-85, 161
November	1,708	1,680	1,566, 1,802	28	-94, 142
December	1,696	1,664	1,550, 1,787	32	-91, 146
Aug-Dec	8,960	8,684		276	
Fall					
August	94	89	66, 117	6	-23, 28
September	83	85	64, 114	-2	-31, 19
October	79	80	60, 106	-1	-27, 19
November	91	74	55, 99	17	-8, 36
December	66	74	55, 99	-8	-33, 11
Aug-Dec	413	400		13	
All other methods					
August	127	136	109, 170	-9	-43, 18
September	133	129	103, 160	4	-27, 30
October	130	130	104, 163	-0	-33, 26
November	118	116	93, 146	2	-28, 25
December	114	110	88, 137	4	-234, 26
Aug-Dec	622	621		1	

Abbreviations: PI, prediction interval

**Fig 4:**
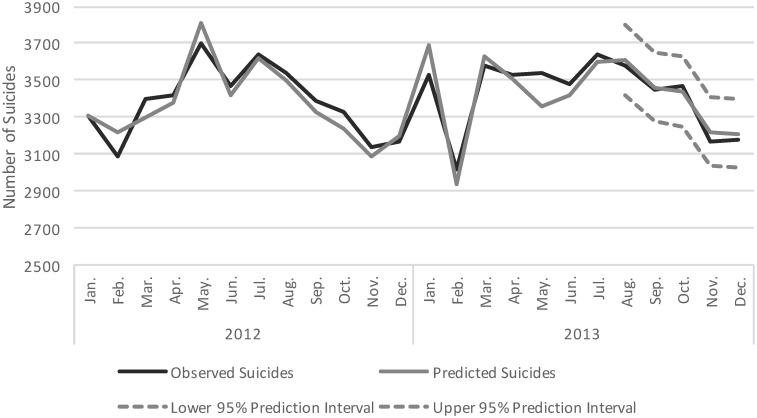
Sensitivity analysis: Observed and predicted number of suicides in the United States from January 2012 to December 2013. Number of observed suicide deaths (black line) and number of predicted suicide deaths (grey line) with 95% prediction intervals (dashed grey lines) are presented.

As a sensitivity test, we used the same method to forecast the five months interval from August to December in 2013, one year prior to Williams’ suicide (Fig 4). Using the same method that we employed to predict the number of monthly suicides following Williams’ death, we found the model performed very well (i.e., the predicted counts overlapped the observed counts), suggesting that any differences between the observed and predicted number of suicides in the months that followed Williams’ death were likely not attributed to modelling decisions.

## Discussion

There was an excess of approximately 1,841 suicides in the United States in the four months after the death of Robin Williams compared to what would be expected for that time period based on forecasted models. While excess suicides were observed across all gender and age groups, the highest excesses were observed for men (1,398 excess suicides) and persons 30–44 years (577 excess suicides). Furthermore, the difference between the observed and expected number of suicides were a function of a greater than expected number of suffocation suicides, over all other methods. Although we cannot determine with certainty that these deaths are attributable to the death of Robin Williams, we found both a rapid increase in suicides in August 2014, and specifically suffocation suicides, that paralleled the time and method of Williams’ death, and a dramatic increase in news media reports on suicides and Robin Williams during this same period, suggesting a connection between Williams’ death and the subsequent increase in suicide deaths from August to December 2014.

Our findings extend previous studies that have documented a parallel increase in media reporting of suicides and suicide rates [1, 6, 15, 18, 19]. Our study used a SARIMA model to estimate the predicted number of suicides in the months that followed Williams’ death. This method represents a substantial improvement over previous methods employed in this literature. For example, Phillips [20], in an attempt to estimate the number of suicides expected in a given month, averaged the number of suicides that occurred in that month during the year prior to and after the event. Whereas our sensitivity analysis demonstrated accurate prediction of suicides from August to December in 2013, one year prior to Williams’ suicide, Phillips [20] averaging method overestimated the predicted number of suicides in August 2014, predicting 3746 suicide deaths in August 2014 (3580 deaths in August 2013 plus 3912 deaths in August 2015 divided by 2), compared to our SARIMA model, predicting 3585.72 (95% CI: 3399.25, 3782.42). Increased access to fast, low-cost, computing over the past several decades has led to more recent literature using SARIMA models to predict suicide deaths in a given month, over Philips averaging method.

Our findings that document a parallel increase in media reporting of suicides and suicide rates suggest that media reporting possibly inducing changes in suicidal ideation via reinforcement, as social learning theory suggests [21, 22]. In this regard, Kumar et al. (2015) described how Williams’ death was also followed by an increased number of online posts in the “SuicideWatch” forum (a suicide support platform in Reddit), and also with changes in posted content linked to suicidal ideation (e.g. expressing greater negativity, anxiety and anger, and manifesting decreased social concerns) [23]. Further, it is hypothesized that celebrity suicides may influence the population patterns of suicide given the ubiquity of knowledge about the celebrity, and potential identification with the celebrity as a model for a subsequent suicide. Identification with the deceased as a mechanism for so-called “copycat suicides” has long been described as a “Werther effect” [20, 24], named after a 1774 novel by Johann Wolfgang von Goethe. That we observed the greatest excess suicide cases were among persons who used a similar method (i.e., suffocation) and who were of the same sex (i.e., male) and in a similar age group as Williams, it is possible that this excess in suicide cases is an example of the “Werther effect”.

The link between media and suicides is also supported by the fact that interventions aiming at guiding media reporting have an impact on suicides following specific suicide cases [1, 15, 25, 26]. Popular news media headlines suggest that media guidelines for suicide reporting were not followed in the case of Mr. Williams. For example, media guidelines suggest that explicit description of the suicide method be avoided, as well as speculation on causes or details of site of suicide, especially in headlines [14]; however, on August 12^th^, 2014, the Washington Post reported “Robin Williams’s death shows the power of depression and the impulsiveness of suicide” [27] and the New York Times reported with the headline “Robin Williams Died by Hanging, Official Says” [28]. Similar headlines can be found across many other population news media sources. A public news conference by the Sheriff assigned to the case detailed the belt that was used in Williams’ death, his body position, and wrist marks, among other details. Thus, substantial evidence suggests that the major US news media outlets tended to deviate from the established suicide reporting guidelines following Williams’ death.

Oversights in media reporting regarding the death of Robin Williams stands in contrast to another high profile US entertainment star suicide, Kurt Cobain, lead singer of the enormously popular rock band Nirvana, in 1994. There was minimal impact of Cobain’s death on suicide rates in the Seattle area of the United States, and available evidence indicates that restrictive reporting of the details of death, as well as consistent messages regarding suicide prevention throughout reporting, may have played a pivotal role in preventing subsequent suicides [29, 30]. It should be noted that this study only counted suicides before and after Cobain’s death in the Seattle area (29), and it is possible that the suicide trends in Seattle differed from the national trends.

It is also worth considering the role of social media as a new and emerging risk factor for how information is disseminated after the death of a celebrity. The PEW research center estimates that as of 2015, approximately 65% of adults in the US receive their news updates through Facebook and Twitter [31]. Even if traditional media outlets follow established reporting guidelines for celebrity suicides, the role of social media speculation and news dissemination may counter any prevention messages that are disseminated in traditional sources.

There are 4 limitations important for interpretation of this study. First, we cannot determine with certainty that the increase in deaths from August to December 2014 can be attributed to the death of Robin Williams. It is possible that a different event, capable of increasing population suicide rates, occurred during the same period as Williams’ death in August 2014. While we cannot entirely rule-out such an event during this time period, we find this hypothesis unlikely given that the greatest spike in suicide-related media reports occurred on the days following Williams’ death. Second, we did not model exogenous factors that could increase suicide risk in the population (e.g., unemployment, weather patterns). However, the SARIMA model used data from 204 time points to model the mechanism generating the monthly suicide deaths in the U.S., without the need for input from exogenous factors. However, correct specification of a SARIMA model can produce accurate predictions without the need for modeling exogenous factors. To verify our model specification, we used a sensitivity analysis to accurately predict suicide counts during the same time period (August to December) in 2013 (i.e., the year prior to Williams’ death), providing evidence accurate model specification without a need to consider exogenous factors. Third, the length of time that media reports influence copycat suicides is unknown. While we modeled a 4 month copycat effect following Williams’ suicide, many previous studies have assumed that the impact of media reporting on copycat suicides lasts approximately 4 weeks after the event [4, 9–11]. Two factors influenced our decision to consider this length of time: (1) monthly suicide counts did not return to pre-Williams’ levels until December 2014 and (2) media reports continued for several months after Williams’ death. Finally, misclassification of suicides in the death records might have led to underestimates of the effects.

Our work adds evidence about the relationship between celebrity suicide, the subsequent media coverage of the event, and subsequent suicide deaths. The number of excess deaths was greatest among men and persons 30–44 years, while suffocation deaths increased over all other suicide methods. Although we cannot determine with certainty that the excess suicides were attributable to news media reports on Williams’ death, Williams’ death might have provided the necessary stimulus for high-risk segments of the U.S. population (e.g., middle-aged men in despair) to move from suicidal ideation to attempt. Therefore, the media industry can positively or negatively influence imitation suicides. Suicide remains a central threat to public health, and high profile celebrity suicides will continue to occur; preventing such effects will require substantial resources and training, as well as creative responses to emerging media.
